# Triceps Surae Short Latency Stretch Reflexes Contribute to Ankle Stiffness Regulation during Human Running

**DOI:** 10.1371/journal.pone.0023917

**Published:** 2011-08-22

**Authors:** Neil J. Cronin, Christopher P. Carty, Rod S. Barrett

**Affiliations:** Musculoskeletal Research Program, School of Physiotherapy and Exercise Science, Griffith University, Queensland, Australia; University of Alberta, Canada

## Abstract

During human running, short latency stretch reflexes (SLRs) are elicited in the triceps surae muscles, but the function of these responses is still a matter of controversy. As the SLR is primarily mediated by Ia afferent nerve fibres, various methods have been used to examine SLR function by selectively blocking the Ia pathway in seated, standing and walking paradigms, but stretch reflex function has not been examined in detail during running. The purpose of this study was to examine triceps surae SLR function at different running speeds using Achilles tendon vibration to modify SLR size. Ten healthy participants ran on an instrumented treadmill at speeds between 7 and 15 km/h under 2 Achilles tendon vibration conditions: no vibration and 90 Hz vibration. Surface EMG from the triceps surae and tibialis anterior muscles, and 3D lower limb kinematics and ground reaction forces were simultaneously collected. In response to vibration, the SLR was depressed in the triceps surae muscles at all speeds. This coincided with short-lasting yielding at the ankle joint at speeds between 7 and 12 km/h, suggesting that the SLR contributes to muscle stiffness regulation by minimising ankle yielding during the early contact phase of running. Furthermore, at the fastest speed of 15 km/h, the SLR was still depressed by vibration in all muscles but yielding was no longer evident. This finding suggests that the SLR has greater functional importance at slow to intermediate running speeds than at faster speeds.

## Introduction

The stretch reflex is a neurophysiological response to the stimulation of muscle spindles. In the human triceps surae muscle group, the stretch reflex can be broadly divided into 3 components based on their onset latencies. The earliest response, the short latency reflex (SLR), is predominantly mediated by proprioceptive information from velocity-sensitive muscle spindle Ia afferents, but may also receive inputs from other sensory receptors [1] and from the motor cortex [2]. During unconstrained human walking, SLR responses may not be naturally elicited in triceps surae muscles, but during the faster movement of running, the SLR has been observed in the gastrocnemius and soleus muscles as a short-lasting burst of activity superimposed on the pre-programmed activity that starts prior to ground contact [3], [4]. The size of the SLR dramatically decreases in the presence of ischemia, which is known to suppress Ia afferent activity [3], [5], confirming the major contribution of the Ia pathway to the SLR.

The precise function of the SLR during locomotion is a matter of ongoing study. It has been proposed on the basis of cat data that the SLR compensates for transient decreases in muscle stiffness in response to a joint perturbation [6], thereby minimising muscle yielding [7]. In humans, evidence has been presented in support of this hypothesis in seated conditions [7], [8]. However, studies of SLR responses during the more complex task of human running have focused on the existence, timing and responsible neural pathways [3], [4], while the functional importance of the SLR is largely unexplored. In order to examine this issue during running, a method is required that can modify the size of the SLR. One potential method is high frequency Achilles tendon vibration, which decreases the efficacy of Ia afferent activity [9], [10]. Although muscle spindle type II and Golgi tendon organ (GTO) Ib afferents are also influenced by this method, they are much less sensitive to vibration than Ia afferents [9], [10], [11], [12]. Tendon vibration decreases SLR amplitude in the human soleus muscle in response to a rapid dorsiflexion perturbation during standing [13], [14], sitting [7] and walking [15], and may thus be a suitable method of suppressing the SLR in running.

The purpose of this study was to examine triceps surae SLR function at different running speeds using Achilles tendon vibration to modify SLR size. Two hypotheses were tested: 1) High frequency Achilles tendon vibration would decrease SLR size in triceps surae muscles during running; 2) Assuming that the SLR is important for muscle stiffness regulation, a vibration-induced decrease in reflex amplitude, if sufficiently large, would lead to evidence of yielding at the ankle joint.

## Materials and Methods

### Participants

Ten healthy participants (7 males, 3 females; age 26±4 years; height 178±9 cm; body mass 71±12 kg) with no history of neurological, cognitive, metabolic, cardiovascular, pulmonary or lower limb musculoskeletal impairment volunteered to participate in this study. Prior to testing, participants were fully informed of the experimental procedures, and each participant provided written informed consent. The study was approved by the Griffith University Human Research ethics committee, and was performed in accordance with the Declaration of Helsinki.

### Protocol

Participants initially ran on an instrumented split-belt treadmill at a speed of 10 km/h for 2 minutes for familiarisation. Participants then ran at 7, 10, 12 and 15 km/h in a randomised order, with rest periods of 3–5 minutes between each speed to avoid fatigue. Two Achilles tendon vibration conditions were assessed at each speed: no vibration and 90 Hz vibration. Vibration was applied unilaterally to the right leg. Prior to data collection participants ran at each speed for a minimum of 30 s to enable adaptation to the speed. Subsequently, 30–40 s of data were collected for each vibration condition to allow meaningful data averaging. At each speed, the no vibration condition was always performed first to avoid after-effects of vibration that can last for several seconds [16]. A minimum of 15–20 s elapsed between conditions. Participants were instructed to look forwards at all times to avoid possible effects of altered head and neck orientation on vestibulo/corticospinal-induced motoneurone excitability [17] or neck proprioceptive input [18].

### Data collection and analysis procedures

#### Electromyography (EMG)

Surface EMG activity was recorded using bipolar surface electrodes (Duo-trode, Myotronics Inc; Australia) with an inter-electrode distance of 2 cm. Data were collected telemetrically (Noraxon Telemyo; AZ, USA) from the soleus (Sol), medial gastrocnemius (MG), lateral gastrocnemius (LG) and tibialis anterior (TA) muscles of the right leg at 1 kHz. EMG signals were band-pass filtered (10 Hz–500 Hz), rectified, low-pass filtered (40 Hz) and ensemble averaged to produce EMG profiles. Mean background EMG was quantified as the mean EMG throughout the stance phase.

To calculate SLR onset latencies, mean EMG was first calculated over the first 30 ms of the contact phase, i.e. prior to the commencement of the SLR which is approximately 40 ms at the earliest [19], [20], [21]. Onset latency was then determined as the first time point where this mean value was exceeded by 4 standard deviations. Visual inspection of all traces suggested that this procedure provided accurate and consistent latency estimates. SLR amplitude was defined as the difference between the level of activity at SLR onset and the size of the subsequent peak, thus taking into account differences in EMG activity at SLR onset between speeds [3], [4].

#### Kinematics and ground reaction forces (GRFs)

Kinematics of the pelvis and right leg were recorded using a 4 camera 3D motion analysis system (Vicon, Oxford Metrics; Oxford, UK) sampling at 100 Hz. Reflective markers were placed in accordance with the lower body model of Besier et al. [22]. A marker was also placed on the vibrator to track displacement of the device relative to the ankle joint centre for each condition. Knee and ankle joint angles were determined from inverse kinematic analysis of the marker trajectories using Opensim software [23]. GRFs were recorded separately from each leg at 1 kHz using 8 triaxial force sensors embedded in the split-belt treadmill (Bertec; OH, USA). At each speed and for each condition, steps were only included in the analysis if step duration was within ±5% of the averaged step duration for that condition, as determined from the treadmill GRF signals. This resulted in the inclusion of 32±2 steps per condition across all speeds. All reported EMG and GRF values were expressed relative to their respective peak values at 7 km/h.

#### Ankle yielding

Ankle and GRF difference curves were computed between the control and vibration mean traces over the time period 55–150 ms after ground contact, as this interval incorporates the expected time course of ankle yielding due to SLR depression [7], [24]. Ankle yielding was defined as a deviation in ankle trajectory between the control and vibration traces, and the amplitude and slope of this deviation were computed.

#### Achilles Tendon vibration

**Figure 1:**
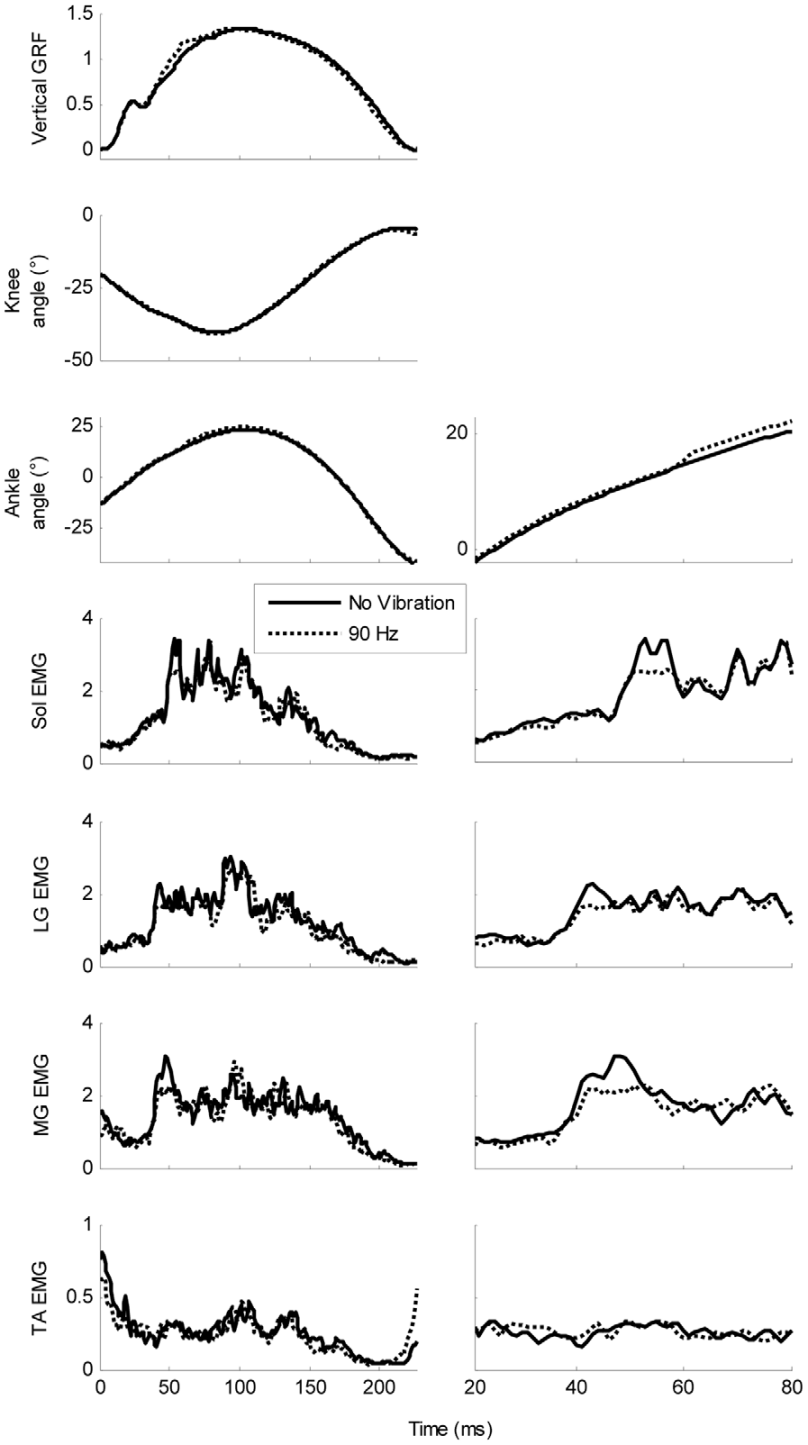
Data from a representative participant running at 12 km/h. Left: GRF, kinematic and EMG data (data averaged from 35–37 steps per condition). Right: EMG traces for all muscles and ankle joint angle shown on an enhanced timescale. For the sake of clarity, ankle angle is also shown on an enhanced scale on the y-axis.

A servo-controlled custom-made vibrating motor (35×25 mm; 100 g) was attached over the Achilles tendon of the right leg using compressive tape designed to be sufficiently compliant to prevent blood flow occlusion but rigid enough to minimise movement. The motor was positioned approximately 3 cm proximal to the ankle joint. High frequency vibration was applied at 90 Hz as this frequency has been shown to produce optimal SLR depression [14], [25]. The motor was switched on approximately 5 s before data collection, remained on for the 30–40 s recording window, and was then switched off. Data from a typical participant running at 12 km/h are shown in Figure 1.

### Statistical analysis

Two-way repeated measures ANOVA was used to assess the effects of vibration condition (no vibration, 90 Hz vibration) and locomotion speed (7, 10, 12, 15 km/h) on all outcome measures. Mauchly's test of sphericity was used to test the assumption of sphericity. Where this assumption was violated, Geisser-Greenhouse (GG) adjustments were used. Where significant main effects were observed, pair-wise comparisons were used to identify the location of differences between vibration conditions at each speed. For all tests, the minimum level of statistical significance was set at p<0.05.

## Results

### Short latency reflex responses

**Figure 2:**
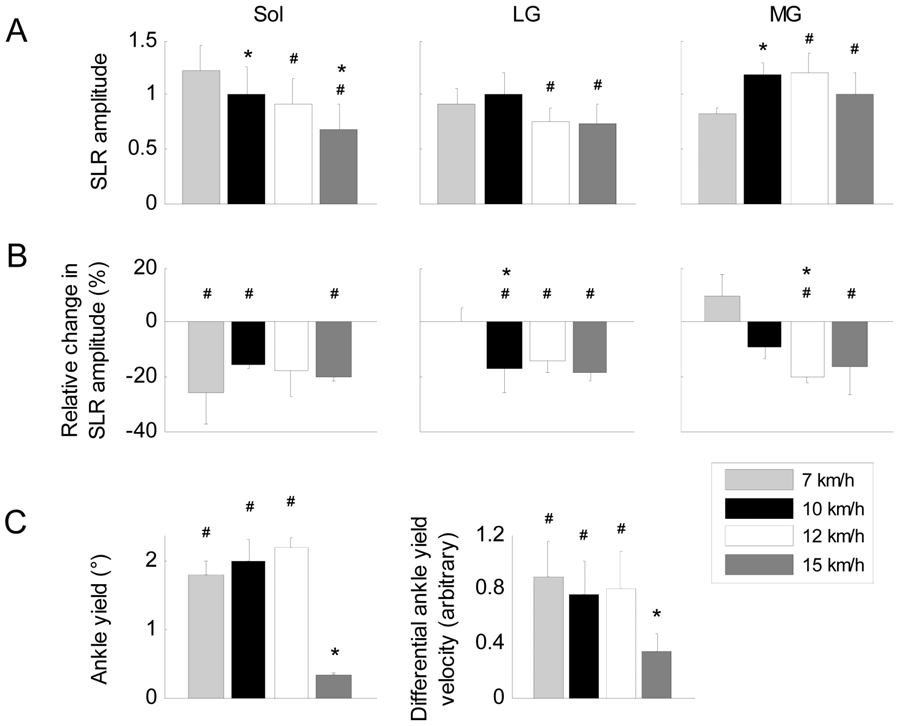
Mean SLR data. A: Mean SLR amplitude at all speeds in the soleus and gastrocnemius muscles. B: Mean relative changes in SLR amplitude due to Achilles tendon vibration. C: Amplitude and velocity of ankle yielding at all speeds computed as the difference between the no vibration and 90 Hz vibration conditions 55–150 ms after ground contact. Positive values denote yielding in the 90 Hz vibration condition. For all plots, * denotes a significant difference from the immediately preceding running speed, and ^#^ denotes a significant difference between the no vibration and 90 Hz vibration conditions at a minimum level of p<0.05. Vertical bars represent 1 SD of the mean. n = 10 for all plots.

Running speed had a significant main effect on SLR amplitude in all triceps surae muscles (soleus: F_2, 18_ = 1.36, p<0.001; medial gastrocnemius: F_2, 18_ = 6.39, p<0.001; lateral gastrocnemius: F_2, 18_ GG = 5.03, p<0.01; Figure 2A). There was also a main effect of vibration on SLR amplitude in soleus (F_2, 18_ = 7.656, p<0.05), LG (F_2, 18_ GG = 5.120, p<0.05) and MG (F_2, 18_ = 8.434, p<0.05; Figure 2B). The effects of these changes on ankle joint kinematics are shown in Figure 2C. Neither running speed nor vibration had a significant main effect on SLR latency in any of the examined muscles (soleus: F_2, 18_ = 0.97–1.05, p = 0.426–0.563; medial gastrocnemius: F_2, 18_ GG = 0.57–0.74, p = 0.651–0.701; lateral gastrocnemius: F_2, 18_ = 0.404–0.632, p = 0.558–0.753).

### Background muscle activation

During running at 7 km/h, average stance phase EMG decreased in Sol due to 90 Hz vibration (F_2, 18_ = 5.51, p = 0.017), but was not statistically altered by vibration at all other speeds (F_2, 18_ = 5.51, p = 0.100–0.938). Vibration had no statistically significant effects on average EMG in LG (F_2, 18_ = 1.09, p = 0.365), MG (F_2, 18_ = 0.10, p = 0.908) or the antagonist TA (F_2, 18_ GG = 1.06, p = 0.347). Average EMG was also computed in the period of stance that preceded the SLR response (approximately 0–40 ms, depending on SLR onset). No statistical differences were observed between conditions in Sol (F_2, 18_ = 7.70, p = 0.726), LG (F_2, 18_ GG = 1.39, p = 0.348) or MG (F_2, 18_ = 6.60, p = 0.405). Furthermore, no changes were evident in the TA muscle in the 0–40 ms time window (F_2, 18_ GG = 0.22, p = 0.685).

### Global effects of vibration on kinematics and GRFs

Vibration had no statistically significant effects on any kinematic or GRF parameters when compared over the entire stance or step cycle. These included the range of ankle joint rotation during the stance phase (F_2, 18_ = 0.46, p = 0.645) and the entire step cycle (F_2, 18_ GG = 3.40, p = 0.100); ankle angle at ground contact (F_2, 18_ = 1.33, p = 0.300); range of knee joint rotation during stance (F_2, 18_ = 1.56, p = 0.257) and the entire step cycle (F_2, 18_ GG = 0.20, p = 0.694); knee angle at ground contact (F_2, 18_ = 1.37, p = 0.291); the area under the vertical GRF curve (F_2, 18_ = 4.60, p = 0.926) and peak vertical GRF (F_2, 18_ GG = 0.08, p = 0.830). Regardless of speed, the duration of the stance phase (F_2, 18_ = 3.63, p = 0.350) and the entire step cycle (F_2, 18_ GG = 1.28, p = 0.295) were both statistically unaffected by vibration.

### Local effects of vibration on kinematics and GRFs

Vibration-induced depression of SLR amplitude in the triceps surae muscles led to ankle yielding starting approximately 60 ms after ground contact and lasting approximately 80 ms. There was a main effect of both vibration condition (F_2, 18_ = 3.128, p<0.05) and running speed (F_2, 18_ = 2.847, p<0.05) on the amplitude of ankle yielding. There was also a main effect of vibration condition (F_2, 18_ GG = 4.430, p<0.05) and running speed (F_2, 18_ GG = 20.826, p<0.01) on the velocity of ankle yielding. There was no main effect of vibration condition (F_2, 18_ = 0.338, p = 0.577) or running speed (F_2, 18_ = 2.113, p = 0.125) on yielding in the vertical GRF trace. Mean SLR and ankle yield data are shown in Figure 2.

### Vibration efficacy

The mean range of displacement of the vibrating motor relative to the ankle joint centre ranged between 0.93±0.25 and 3.17±2.0 mm. Neither vibration condition (F_2, 18_ = 1.19, p = 0.345) nor gait speed (F_2, 18_ = 1.48, p = 0.141) significantly influenced the range of vibrator displacement. To determine whether the effects of vibration were altered over the course of a trial, the level of SLR depression due to vibration was compared between the first and last 5 steps of each 90 Hz vibration trial. Across all speeds there was no main effect in Sol (F_2, 18_ = 0.37, p = 0.812), LG (F_2, 18_ = 1.03, p = 0.528) or MG (F_2, 18_ = 0.78, p = 0.611).

## Discussion

This study sought to examine the function of the short latency stretch reflex in human triceps surae muscles during running, using tendon vibration to modify the strength of the reflex responses. In support of our hypotheses, vibration produced clear decrements in triceps surae SLR size at all of the examined speeds. At running speeds between 7 and 12 km/h, where SLR amplitude was largest, vibration-induced depression of the SLR led to yielding at the ankle joint, suggesting that the SLR contributes to muscle stiffness regulation during running by minimising ankle yielding during the early contact phase. At the fastest running speed of 15 km/h, where the SLR was generally at its smallest, vibration still clearly decreased SLR size in all muscles, but ankle yielding was no longer evident. This suggests that the SLR plays a more important functional role at slow to intermediate running speeds than at faster speeds.

### The origin of short latency stretch reflexes in running

A fundamental issue in this study is whether the SLR responses observed here are genuine reflex responses or the result of a sudden increase in pre-programmed activity. In running, Dietz et al. [3] reported bursts of activity at latencies as early as 30–40 ms after ground contact in the medial gastrocnemius muscle. These responses were attributed to short latency reflexes since ischemia clearly suppressed the size of the EMG burst. In contrast, Ishikawa and Komi [4] reported considerably longer, speed-dependent onset latencies in the medial gastrocnemius but did not attempt to verify the reflexive nature of the responses. Our data using tendon vibration to suppress the EMG burst are consistent with the findings of Dietz et al. [3], suggesting that SLRs occur approximately 40 ms after ground contact in triceps surae muscles during running, and that their onset latencies are unaffected by speed.

The discrepancy in the literature regarding SLR latencies raises further questions concerning the stimulus for the SLR. Ishikawa and Komi [4] attributed the SLR to a rapid stretch of the muscle fascicles following foot contact, based on the observation that the time between fascicle stretch onset and SLR onset was constant at different running speeds. However, muscle fascicle stretch commenced 21–37 ms after ground contact. This would occur too late to trigger an SLR with a latency of 40 ms, as this value corresponds to the shortest possible SLR latency in these muscles [21]. The latencies observed here and by Dietz et al. [3] are consistent with the alternative view that the stimulus for the SLR is the propagation of mechanical vibration that results from foot-ground contact, and subsequently excites muscle spindles. In response to an Achilles tendon tap or foot sole vibration, SLRs are evoked in triceps surae muscles [1], as well as in more proximal leg muscles not exposed to stretch [25], [26]. The SLR can even be elicited during muscle shortening, further suggesting that the response is not triggered by fascicle stretch [1], [26], although this does not necessarily preclude an effect of fascicle stretch parameters on later reflex components. We attribute the stimulus of the SLR responses observed in this study to the transmission of mechanical vibration beginning at foot-ground contact, which can account for the consistent SLR latencies of approximately 40 ms observed here. A similar hypothesis has been proposed to account for SLR responses evoked during stumbling over an obstacle [27].

### The functional importance of the SLR during running

When an isometrically contracting muscle is rapidly stretched, the amplitude of the resulting SLR is generally largest when intrinsic muscle stiffness is low, and thus muscle yielding is more likely to occur [28], [29]. Conversely, as muscle force increases, intrinsic stiffness also increases, so the likelihood of muscle yielding in response to the same stretch stimulus decreases, resulting in a smaller SLR [6], [7], [29], [30], [31]. Data from the present study provide support for this hypothesis in running, as vibration-induced depression of SLR responses coincided with high velocity ankle yielding at running speeds between 7 and 12 km/h, where SLR amplitude was largest. At the fastest running speed of 15 km/h, where intrinsic muscle stiffness would be expected to be higher, vibration still depressed the SLR in all muscles but ankle yielding was not observed. The SLR was also generally smallest at this speed. These findings suggest that the functional importance of the SLR declines at fast running speeds in this muscle group, as is the case at high force levels in isometric conditions.

Yielding at a joint may have significant functional implications during locomotion. For example, in cats, weakening of the ankle extensors by denervation of certain muscles leads to dramatic yielding at the ankle that cannot be immediately compensated for, resulting in severe disruption of the kinematic patterns at the ankle and knee [32], [33]. Yielding would also be expected to lengthen the muscle fascicles, which could alter the force-generating potential of the muscle and thus influence locomotor energetics. With regard to long-term implications, changes to the nervous system during development and after injury or training would require adaptation of reflex input in order to maintain optimal motor output and minimise yielding during locomotion [33].

### Mechanisms of vibration-induced SLR depression during running

It is well established that the velocity-sensitive Ia pathway makes an important contribution to the SLR. Tendon vibration generally exerts its most potent effects on Ia afferents, which are more sensitive to vibration than type II or Ib fibres [34]. Accordingly, vibration led to clear suppression of SLR responses in this study. However, several other pathways may contribute to the SLR including Ib afferents, cutaneous receptors and mechanoreceptors in other muscles [1], as well as a potential role of pre-programmed input from the motor cortex [2]. As the vibrating motor was switched on several seconds before data collection began, vibration may have suppressed ongoing activity from sensory receptors such as spindle type II and cutaneous afferents, which could in turn have modified the net Ia input to the motoneurones or the excitability of the motoneurones directly. Nonetheless, it is noteworthy that tizanidine (a selective group II afferent inhibitor) and lidocaine (a cutaneous afferent inhibitor), both of which require a longer time frame than vibration to take effect, do not influence mechanically evoked SLRs during locomotion [19]. The relative contribution of each of the pathways contributing to the SLR may change at different running speeds and in different muscles. Therefore, the observed patterns of SLR modulation are not simply a reflection of changes in Ia afferent activity.

### Methodological considerations

In some participants we observed a transient fluctuation in vibration frequency of approximately ±5 Hz shortly after ground contact. Changes of such small magnitude are unlikely to affect vibration efficacy, as vibration frequencies between 70–100 Hz have been shown to decrease Ia-mediated responses [7], [10], [35]. Previous studies have shown that unilateral Achilles tendon vibration has no effects on joint displacements of the non-vibrated limb, suggesting that intra-limb coordination is unaffected by this paradigm, at least during walking [36]. It should be noted that the amplitude of ankle yielding observed at running speeds between 7 and 12 km/h may have been substantially greater if a more potent Ia afferent blocking technique, such as ischemia, was used. We elected not to use this method because of its numerous limitations including the time required to induce ischemia, the limited time frame allowed for data collection and pain in the affected limb (see [37]). Using vibration we observed peak ankle yielding of 2.3°, which represents approximately 5% of the ankle range of motion during slow running. Allum et al. [7] reported clearer evidence of yielding in seated conditions, although in their study vibration produced SLR decrements of up to 80%, which is much larger than the values obtained in this study. Yielding is also the resultant effect of changes in all plantar flexor muscles, several of which were not examined in this study. It is therefore likely that our data underestimate the extent of yielding that would occur in the absence of SLR activity, and thus the functional importance of the SLR.

### Conclusions

During human running, SLR responses have long been known to occur in triceps surae muscles, but their functional relevance has not been determined in this context. The results of the present study showed that suppression of predominantly Ia afferent-mediated SLR responses using Achilles tendon vibration led to evidence of ankle yielding at slow to intermediate running speeds, but not at the fastest speed of 15 km/h. These results provide strong evidence for a role of the SLR in ankle stiffness regulation during the early contact phase of human running. In addition, our results suggest that the functional importance of the SLR in triceps surae muscles is speed-dependent, being greater at slow to intermediate running speeds than at faster speeds.
